# Caracterización de exodoncia de terceros molares

**DOI:** 10.21142/2523-2754-0903-2021-075

**Published:** 2021-10-06

**Authors:** Solange Baeza, Nathaly Cáceres, Gustavo González, Carolina Guzmán, María Paz Sepúlveda, Isidora Valenzuela

**Affiliations:** 1 Cátedra de Cirugía y Traumatología Oral y Maxilofacial, Facultad de Odontología, Universidad de Valparaíso. Valparaíso, Chile. solange.baeza@uv.cl Universidad de Valparaíso Cátedra de Cirugía y Traumatología Oral y Maxilofacial Facultad de Odontología Universidad de Valparaíso Valparaíso Chile solange.baeza@uv.cl; 2 Facultad de Odontología, Universidad de Valparaíso. Valparaíso, Chile. nathaly.caceres@alumnos.uv.cl, gustavogonzalezgratz@gmail.com, carolina.guzman@alumnos.uv.cl, maria.sepulveda@alumnos.uv.cl, isidora.valenzuela@alumnos.uv.cl Universidad de Valparaíso Facultad de Odontología Universidad de Valparaíso Valparaíso Chile nathaly.caceres@alumnos.uv.cl gustavogonzalezgratz@gmail.com carolina.guzman@alumnos.uv.cl maria.sepulveda@alumnos.uv.cl isidora.valenzuela@alumnos.uv.cl

**Keywords:** extracción dental, tercer molar, protocolos clínicos, cirugía oral, tooth extraction, third molar, clinical protocols, oral surgery

## Abstract

El objetivo de este estudio fue caracterizar las principales situaciones de indicación, contraindicación y accionar profiláctico para la exodoncia de terceros molares, según la literatura. Se utilizaron las bases de datos PubMed, Scopus y Web of Science, con un total de 3 llaves de búsqueda, y se incluyeron publicaciones de máximo 5 años de antigüedad, en inglés o español, con disponibilidad de texto completo. Fueron seleccionados 18 artículos, en los que se determinó que la indicación más frecuente en la exodoncia de terceros molares fue el diagnóstico de pericoronaritis, seguido de caries cervical distal en segundo molar inferior, reabsorción radicular en dientes adyacentes y quistes. A su vez, puede estar indicada para facilitar otros tipos de tratamientos dentales, como ortodoncia, cirugía ortognática y rehabilitación. En cuanto a las contraindicaciones, predominó el riesgo de complicaciones intraoperatorias asociadas con el nervio alveolar inferior, edad avanzada y compromiso sistémico. El 72,2% de los artículos incluía en su análisis la extracción profiláctica, y se observa una predominancia de exodoncia de terceros molares asintomáticos y libres de enfermedad. Finalmente, la evidencia señala situaciones claras de indicaciones y contraindicaciones en torno a la exodoncia de terceros molares. Estas se asocian, principalmente, con cambios patológicos, indicaciones por tratamientos de ortodoncia, cirugía ortognática y tratamientos rehabilitadores. Sin embargo, existe controversia en la literatura respecto de la exodoncia profiláctica, y se destaca que no todos los terceros molares retenidos y asintomáticos deben extraerse. Es necesario evaluar caso por caso, considerando los beneficios de la práctica, las características del paciente y el riesgo de complicaciones posoperatorias.

## Abstract (en)

The aim of this study was to characterize the main indications, contraindications and prophylactic actions for third molar extraction according to the literature. The Pubmed, Scopus and Web of Science databases were searched using a total of 3 search keys, including publications up to 5 years old, in English or Spanish, with availability of full text. Eighteen articles were selected and showed that the most frequent indication for third molar extraction was the diagnosis of pericoronaritis, followed by distal cervical caries in the lower second molar, and root resorption in adjacent teeth and cysts. In turn, it may be indicated to facilitate other types of dental treatment, such as orthodontics, orthognathic surgery and rehabilitation. In relation to contraindications, the risk of intraoperative complications was associated with the inferior alveolar nerve, advanced age and the predomination of systemic involvement. Prophylactic extraction was included in the analysis in 72.2% of the articles, with a predominance of exodontia of asymptomatic and disease-free third molars. Finally, there are clear indications and contraindications for third molar extraction, being mainly associated with pathological changes, indications for orthodontic treatment, orthognathic surgery and rehabilitative treatment. However, there is controversy in the literature regarding prophylactic extraction, emphasizing that not all retained and asymptomatic third molars should be extracted. Third molar extraction should be evaluated on a case by case basis, considering the benefits, the patient's characteristics and the risk of postoperative complications.

## INTRODUCCIÓN

El tercer molar (3M) es uno de los dientes con mayor variabilidad tanto en morfología como en cronología de erupción ^1^, la cual no suele pasar inadvertida, sino que se asocia con dolor, hinchazón e infección. De esa forma, la exodoncia de terceros molares (3Ms) se ha convertido en una práctica clínica común y en uno de los procedimientos más frecuentes en cirugía bucal ^2^^,^^3^. En consecuencia, la decisión de someter a un paciente a este procedimiento es compleja y desafiante, debido a que ningún profesional desea exponer al usuario al riesgo quirúrgico, incomodidad propia del procedimiento, y la carga financiera que involucra, a menos que se identifique un claro beneficio ^4^. Por lo tanto, el odontólogo general debe ser capaz de indicar correctamente la exodoncia de 3Ms y realizar una derivación oportuna. 

Las indicaciones para exodoncia de 3Ms han quedado sujetas a la evaluación de la correlación entre el desarrollo de pericoronaritis, lesiones quísticas y caries asociadas con dientes retenidos ^5^. Por otro lado, las razones para mantener en boca los 3Ms, según la evidencia disponible, es controversial, debido a las complicaciones que pueden surgir en el futuro, producto de su retención ^6^. Algunos autores han identificado que un tercer molar sin erupción, sin enfermedad y sin síntomas cubierto de hueso, o si el procedimiento de exodoncia constituye un riesgo para la salud general o local del paciente, son razones suficientes para su retención ^7^.

Sin embargo, la extracción de 3Ms sin sintomatología sigue siendo un tema de discusión, pues su extracción profiláctica es una práctica común en odontología y es el principal motivo de indicación de exodoncia para dientes retenidos asintomáticos y libres de enfermedad ^4^. Lo anterior se ha justificado en la probabilidad de desarrollar tumores inflamatorios, quísticos o neoplásicos en el tejido que rodea a estos dientes, sin evidencia clínica o radiográfica de enfermedad ^8^.

A pesar de ser un procedimiento comúnmente realizado, aún existe falta de claridad y unificación en de la evidencia disponible, a lo que se suma un déficit de declaraciones de consenso. Por tanto, el presente estudio tiene como objetivo caracterizar las principales situaciones de indicación, contraindicación y accionar profiláctico, para la exodoncia de 3Ms, según la literatura actualizada. 

## MATERIALES Y MÉTODOS

### Estrategia de búsqueda

Se llevó a cabo una exhaustiva búsqueda de manera sistemática, durante los meses de marzo y abril, en las bases de datos PubMed, Scopus y WOS, mediante los buscadores Google Chrome y Mozilla Firefox, utilizando palabras claves como: “exodontia”, “dental extraction”, “tooth extraction”, “surgery”, “oral surgery”, “third molar”, “wisdom teeth”, “indications”, “recommendations”, “protocols”, “contraindications”, “prophylactic tooth extraction” y “third molar”. Se estructuraron 3 llaves de búsqueda, una para cada base de datos, a partir de diferentes filiales, mediante la ayuda de operadores booleanos como OR y AND (Tabla 1). 

**Tabla 1:** Estrategia de búsqueda. Palabras claves utilizadas en las bases de datos WOS, Scopus y Pubmed, con número de publicaciones respectivo.

Filiales	Términos de búsqueda	Resultados
PubMed		
#1	Exodontia [MeSH Terms] OR dental extraction [MeSH Terms] OR tooth extraction [MeSH Terms] OR surgery [MeSH Terms] OR oral surgery [MeSH Terms] OR exodontia [Title/Abstract] OR dental extraction [Title/Abstract] OR tooth extraction [Title/Abstract] OR surgery [Title/Abstract] OR oral surgery [Title/Abstract]	661,069
#2	Third molar [MeSH Terms] OR wisdom teeth [MeSH Terms] OR third molar [Title/Abstract] OR wisdom teeth [Title/Abstract]	2,092
#3	Indications [MeSH Terms] OR recommendation [MeSH Terms] OR protocols [MeSH Terms] OR contraindications [MeSH Terms] OR indications [Title/Abstract] OR recommendation [Title/Abstract] OR protocols [Title/Abstract] OR contraindications [Title/Abstract]	112,637
Llave de búsqueda I	(#1 AND #2 AND #3)	230
Scopus		
Llave de búsqueda II	(TITLE-ABS-KEY (“exodontia” OR “dental extraction” OR “tooth extraction” OR “surgery” OR “oral surgery”)) AND (TITLE-ABS-KEY (“third molar” OR “widsom teeth”)) AND (TITLE-ABS-KEY (“indications” OR “recomendations” OR “protocols” OR “contraindications”)) OR (TITLE-ABS-KEY (“prophylactic tooth extraction” AND “third molar”))	477
Web of Science		
#1	AB=(exodontia) OR AB=(dental extraction) OR AB=(tooth extraction) OR AB=(surgery) OR AB=(oral surgery)	819,798
#2	AB=(third molar) OR AB=(wisdom teeth)	9,621
#3	AB=(indications) OR AB=(recommendations) OR AB=(protocols) OR AB=(contraindications)	1,059,644
#4	AB=(prophylactic tooth extraction) AND AB=(third molar)	26
Llave de búsqueda III	(#1 AND #2 AND #3) OR #4	292

Fuente: Elaborado por los investigadores.

Se obtuvo 999 publicaciones que fueron estudiadas y preseleccionadas de acuerdo con la lectura de sus títulos y *abstracts*, los cuales debían guardar relación con los objetivos del estudio. Cada uno fue analizado por los investigadores de forma independiente para, luego, ser discutidos en conjunto vía remota mediante la plataforma Zoom, con el fin de llegar a un consenso y, con ello, estandarizar la búsqueda. Ante cualquier conflicto que surgiera en el proceso, se procedió a consultar con un experto.

### Criterios de elegibilidad

• Criterios de inclusión. Se consideraron publicaciones de máximo 5 años de antigüedad, es decir, de los años 2017 a 2021; en idioma inglés o español; y con un resumen disponible coherente con el objetivo de la investigación. Además, se incluyeron todos aquellos artículos cuyos diseños de estudio fueran estudios primarios, revisiones sistemáticas y metaanálisis.

• Criterios de exclusión. Se excluyeron los artículos que no tuvieran acceso a texto completo y los reportes de caso, por su bajo nivel de evidencia.

### Variables de estudio

De los artículos incluidos en esta revisión, las variables a analizar fueron: “autor”, “año de publicación”, “tipo de estudio”, “indicaciones de exodoncia de terceros molares”, “contraindicaciones de exodoncias de terceros molares” y “exodoncia profiláctica”.

Para determinar el nivel de evidencia y grado de recomendación de los artículos, se utilizaron las pautas del Oxford Centre for Evidence-Based Medicine.

## RESULTADOS

**Figura 1:**
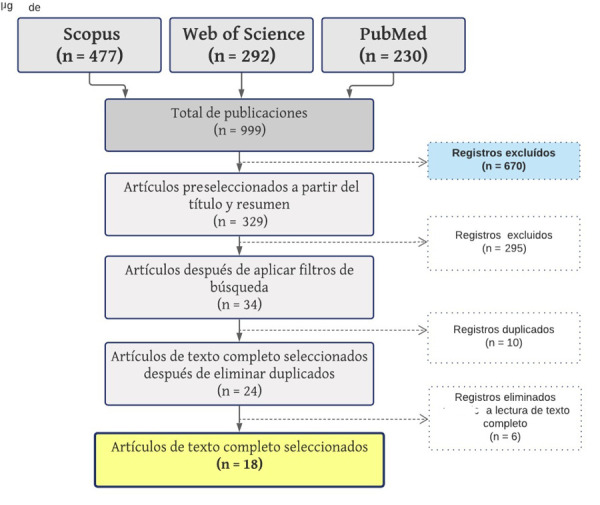
Proceso de selección de artículos

Fueron seleccionados 18 artículos para esta revisión narrativa, según los criterios de elegibilidad establecidos (Figura 1). Se hallaron una revisión sistemática, tres revisiones narrativas, tres estudios de cohorte y once estudios descriptivos. 

A partir de estos resultados, se determinó que el 66,7% de los artículos incluidos en la revisión aluden a la indicación de exodoncia de 3Ms, y predomina la pericoronaritis como la principal causa de extracción (Tabla 2).

**Tabla 2:** Indicaciones para la exodoncia de terceros molares. Artículos incluidos y analizados según autor, año, tipo de estudio, resultados obtenidos como indicación de exodoncia para terceros molares, nivel de evidencia y grado de recomendación.

Autor	Año	Tipo de estudio	Resultados	Nivel de evidencia	Grado de recomendación
McArdle, LW et al.	2018	Estudio de cohorte	Las principales razones para extraer terceros molares fueron pericoronaritis, caries, patologías periapicales (infección periapical) y caries cervical distal del segundo molar inferior. Además es indicado para facilitar tratamientos de ortodoncia y cirugía ortognática.	2b	B
Kim, JY et al.	2017	Estudio descriptivo	Las principales razones para extraer terceros molares fueron pericoronaritis, caries o reabsorción radicular en dientes adyacentes, infección y ampliación del folículo dental.	4	C
Alves-Pereira, D et al.	2017	Estudio descriptivo	Las principales razones para extraer terceros molares fueron pericoronaritis, reabsorción externa y caries distal en el segundo molar en conjunto con el tercer molar en posición horizontal IIIA.	4	C
Wu, YP et al.	2017	Estudio descriptivo	Las principales razones para extraer terceros molares fueron su presencia de forma ectópica y sus patologías asociadas.	4	C
Chitre, IR et al.	2017	Estudio descriptivo	Las principales razones para extraer terceros molares fueron riesgo de impactación asociado a caries, pericoronaritis, defectos periodontales en la superficie distal de los terceros molares y quistes odontogénicos.	4	C
McArdle, LW et al.	2019	Estudio descriptivo	Las principales razones para extraer terceros molares fueron caries cervical distal en 2MI, pericoronaritis, caries y quistes odontogénicos.	4	C
Haidry, N et al.	2018	Estudio descriptivo	Las principales razones para extraer terceros molares fueron presencia de patosis pericoronal, infección, lesiones de caries no restaurables, quistes y tumores.	4	C
Alfadil, L et al.	2020	Estudio descriptivo	Las principales razones para extraer terceros molares fueron pericoronitis, quistes relacionados con la impactación, dolor, infección, caries o si interfieren con tratamientos protésicos u ortodóncicos.	4	C
Smailiene, D et al*.*	2019	Estudio descriptivo	Las principales razones para extraer terceros molares pericoronitis, quistes odontogénicos, tumores, enfermedades periodontales, caries dentales y la reabsorción radicular externa.	4	C
Shoshani-Dror, D et al.	2018	Revisión narrativa	Las principales razones para extraer terceros molares fueron infección, lesiones de caries no restaurables, quistes, tumores, destrucción de dientes y huesos adyacentes.	5	D
Hyam, D et al*.*	2018	Revisión narrativa	Las principales razones para extraer terceros molares fueron caries irrestaurables, patología periapical, pericoronaritis e infección odontogénica, reabsorción radicular, fractura, cirugía ortognática asociada a ortodoncia, tratamiento rehabilitador, resección de tumor y enfermedad periodontal.	5	D
Gray, DG et al.	2017	Revisión narrativa	Las principales razones para extraer terceros molares fueron caries irreversibles, celulitis, absceso, osteomielitis, reabsorción interna/externa del diente o dientes adyacentes y enfermedad del folículo.	5	D

Fuente: Elaborado por los investigadores.

En relación, a las contraindicaciones, el 16,7% de los artículos menciona esta variable en su desarrollo, y predomina el riesgo de complicaciones intraoperatorias, como la lesión del nervio alveolar inferior, con lo que se convierte en una de las principales razones para no extraer los 3Ms (Tabla 3).

**Tabla 3:** Contraindicaciones para la exodoncia de terceros molares. Artículos incluidos y analizados según autor, año, tipo de estudio, resultados obtenidos como contraindicación de exodoncia para terceros molares, nivel de evidencia y grado de recomendación.

Autor	Año	Tipo de estudio	Resultados	Nivel de evidencia	Grado de recomendación
De Bruyn ,L et al.	2020	Estudios de cohorte	Las principales razones para retener los terceros molares fueron erupción hacia la oclusión adecuada, preferencia del paciente de rechazar la extracción sugerida y terceros molares asintomáticos en pacientes mayores de 30 años.	2b	B
Alves-Pereira, D et al.	2017	Estudio descriptivo	Las principales razones para retener los terceros molares fueron riesgo de lesión del nervio alveolar inferior, complicaciones de la cirugía y riesgo de fractura mandibular.	4	C
Shoshani-Dror, D et al.	2018	Revisión narrativa	Las principales razones para retener los terceros molares fueron edades extremas, mayor riesgo de fractura mandibular, acceso quirúrgico deficiente, enfermedad sistémica y mayor riesgo de complicaciones intraoperatorias o posoperatorias.	5	D

Fuente: Elaborado por los investigadores.

El 72,2% de los artículos incluía en su análisis la extracción profiláctica y se observa una predomi-nancia de la exodoncia de 3Ms asintomáticos y libres de enfermedad, como situaciones clínicas en las que se busca evitar el riesgo de complicaciones futuras (Tabla 4).

**Tabla 4:** Exodoncia Profiláctica en terceros molares. Artículos incluidos y analizados según autor, año, tipo de estudio, resultados obtenidos en relación a la exodoncia profiláctica, nivel de evidencia y grado de recomendación.

Autor	Año	Tipo de estudio	Resultados	Nivel de evidencia	Grado de recomendación
Ghaeminia H, et al.	2020	Revisión sistemática	Se recomienda la extracción profiláctica en terceros molares impactados asintomáticos y libres de enfermedad que pueden estar asociados a largo plazo con un mayor riesgo de periodontitis.	1a	A
Shanshan T, et al.	2021	Estudio de cohorte	Se recomienda la exodoncia profiláctica en terceros molares mandibulares mesioangulados, debido a un mayor riesgo de desarrollar patologías asociadas.	2b	B
McArdle, L.	2018	Estudio de cohorte	Se recomienda la intervención profiláctica en pacientes con terceros molares inferiores impactados, con riesgo de causar caries cervical distal del 2MI.	2b	B
L.W.McArdle	2019	Estudio descriptivo	Se recomienda la exodoncia profiláctica frente terceros molares mandibulares mesioangulados (alto riesgo de caries cervical distal en el segundo molar inferior)	4	C
Kim, JY, et al.	2017	Estudio descriptivo	Se recomienda la exodoncia profiláctica en terceros molares impactados con riesgo de patologías que generen daño irreversible y severo en el segundo molar inferior en pacientes jóvenes.	4	C
Alves, D et al.	2017	Estudio descriptivo	Se recomienda la exodoncia profiláctica para prevenir la formación de quistes, cuando existe radiolucidez pericoronal mayor a 2,5 mm.	4	C
De Mello, V.	2018	Estudio descriptivo	Se recomienda la exodoncia profiláctica en terceros molares impactados asintomáticos, ya que se pueden encontrar alteraciones histopatológicas en el folículo como infiltrado inflamatorio y metaplasia escamosa, lo que eventualmente puede generar un quiste odontogénico con el tiempo.	4	C
Smailiene D, et al.	2019	Estudio descriptivo	Se recomienda la intervención profiláctica en terceros molares incluidos con dientes adyacentes que presentan reabsorción radicular.	4	C
Ryalat, S et al.	2018	Estudio descriptivo	La extracción profiláctica de terceros molares asintomáticos se realiza sin criterio estandarizado, esto debido a la dificultad para predecir el impacto que causará si no se extrae.	4	C
Simsek, H et al.	2020	Estudio descriptivo	La extracción profiláctica, no se indica de manera genérica, se debe realizar un seguimiento de rutina para los dientes asintomáticos, que no causan problemas en tejidos circundantes ni subyacentes.	4	C
Hyam, D.M.	2018	Revisión narrativa	Se recomienda la extracción profiláctica en pacientes deportivos o militares, con afecciones neuromusculares, con compromiso cognitivo, premedicados con bisfosfonatos o anticoagulantes, tratamientos previos tales como radioterapia/quimioterapia y con terapias inmunomoduladoras.	5	D
Shoshani, D et al.	2018	Revisión narrativa	La exodoncia profiláctica de terceros molares retenidos,a una edad temprana está justificada porque tienen un alto riesgo de desarrollar diversas patologías. Además, a edades más avanzadas, la extracción se vuelve más compleja, con una mayor tasa de complicaciones, debido al deterioro de las condiciones fisiológicas sistémicas y los cambios en la fisiología ósea.	5	D

Fuente: Elaborado por los investigadores.

## DISCUSIÓN

A partir de los resultados de esta revisión, se informa que la pericoronaritis en su fase crónica es la indicación más común para la extracción del 3M, seguida por la caries cervical distal en segundo molar inferior (2MI), la reabsorción radicular en dientes adyacentes, los quistes odontogénicos, las caries, las patologías periapicales y la enfermedad periodontal. Todas estas pueden generar alteraciones importantes en el desarrollo de actividades cotidianas de los pacientes como deglutir, masticar, hablar e higienizar adecuadamente la cavidad oral ^9^^,^^10^. A su vez, la exodoncia de 3Ms puede estar indicada para facilitar otros tipos de tratamientos odontológicos, como ortodoncia, cirugía ortognática y rehabilitación.

Dichos resultados concuerdan con los de McArdle *et al*. ^11^, quienes mencionan que la pericoronaritis sigue siendo la indicación más común para la eliminación de los 3Ms. Esto puede explicarse por la anatomía local y la presencia de un opérculo de mucosa que recubre la superficie oclusal de las impactaciones verticales y distoangulares, lo que crearía un entorno propicio para infecciones locales de tejidos blandos. Esto, sumado a una higiene bucal deficiente o inadecuada, propiciaría que esta condición sea uno de los problemas más frecuentes con signos y síntomas característicos, como dolor, edema, trismus y halitosis, cuadro que podría agravarse según las características sistémicas del paciente ^3^^,^^12^. Sin embargo, este estudio menciona que la caries del 3M y la caries cervical distal del segundo molar (2M) adyacente, se han vuelto más prevalentes y se observan en grupos de población de mayor edad ^11^. Del mismo modo, estos resultados coinciden con lo encontrado en la literatura. Según De Bruyn *et al*. ^13^, los 3Ms impactados se asocian con cambios patológicos, por lo que, en general, se acepta que la exodoncia del 3M está indicada cuando hay signos y síntomas de enfermedad. 

Por otro lado, existen ciertas indicaciones de exodoncia profiláctica de 3Ms en relación con su posición, tal como lo afirman Alves *et al*. ^14^ al señalar que existe un alto riesgo de generar complicaciones como caries y enfermedad periodontal cuando el 2MI y el tercer molar inferior (3MI) se encuentran en la posición horizontal IIIA, según la clasificación de Pell y Gregory ^15^. Asimismo, la probabilidad de desarrollar caries en la cara distal del 2M aumenta cuando la angulación entre este y el 3M se encuentra entre los 43° y 71°, así como existe el riesgo cuando la distancia entre la unión cemento-esmalte de los dos dientes está entre 3 y 10 mm. Esto coincide con lo que señalan Wu *et al*. ^16^, quienes destacan la importancia de evaluar la posición de los 3Ms de forma clínica y radiográfica, con el fin de prevenir y diagnosticar a tiempo posibles complicaciones, las cuales generarían un impacto no solo en el estado sistémico y de salud del paciente, sino que tendrían consecuencias económicas, por los costos asociados con las intervenciones quirúrgicas. Por tanto, los 3Ms mandibulares ectópicos se deben evaluar precozmente, para tener una indicación de exodoncia de forma absoluta. Cabe destacar que estos suelen encontrarse en pacientes de mediana edad y se ubican generalmente en la rama superior y media de la mandíbula. Esto se contradice con lo señalado por Simsek *et al*. ^17^, quienes recomiendan el seguimiento de rutina para dientes asintomáticos, que no causan problemas significativos en dientes y tejidos adyacentes.

Otra situación clínica en la que se recomienda la exodoncia profiláctica es cuando existe radiolucidez pericoronal mayor a 2,5 mm, con el fin de prevenir la formación de quistes, debido a que aumenta la probabilidad de que surjan cambios patológicos en el folículo dental. En el estudio de Mello *et al*. ^18^, se concluye que las alteraciones histopatológicas se encuentran con mayor frecuencia en pacientes mayores de 20 años. Dicho resultado, se condice con el estudio de Haidry *et al*. ^19^, quienes señalan que existe una incidencia ligeramente mayor de cambios quísticos en el grupo etario de 18 a 24 años, lo que indica que la extracción temprana reduciría la morbilidad quirúrgica y el riesgo de complicaciones posoperatorias.

Sin embargo, hay situaciones clínicas en las cuales existen razones para no extraer los 3Ms, es decir, contraindicaciones para este procedimiento quirúrgico. En primer lugar, se ha establecido, de manera clara, contraindicar la exodoncia en pacientes de edades avanzadas ^2^^,^^13^, debido a que la probabilidad de complicaciones después de la intervención quirúrgica podría ser 1,5 veces mayor que en los pacientes menores de 25 años ^20^. Al respecto, se debe considerar que ha habido una continuidad en el desarrollo de las raíces y el ligamento ha reducido su espesor con el pasar de los años y permitido el desarrollo de anquilosis en algunos casos, lo que dificulta la exodoncia. Además, a medida que el paciente envejece, el hueso presenta mayor densidad producto de su calcificación, lo cual provoca una disminución en la flexibilidad y un aumento en la probabilidad de fractura. Por lo tanto, el hueso debe ser extraído de manera quirúrgica, lo que hace al procedimiento más invasivo y prolonga la recuperación y disminuye su calidad ^21^^,^^22^. En un estudio transversal basado en dentistas españoles y portugueses, se pudo observar concordancia en determinar que el riesgo de fractura de mandíbula y el hecho de que la cirugía fuese demasiado agresiva se catalogarían como contraindicaciones para la extracción de 3Ms ^14^.

En segundo lugar, la adecuada valoración de la salud sistémica del paciente permitirá reconocer condiciones basales que contraindican el ingreso de pacientes a este tipo de procedimientos. Un estudio demostró que el compromiso de la salud impedía de manera significativa la extracción de 3Ms en hombres y mujeres en el rango etario de 50 a 70 años, a diferencia de individuos menores de 30 años ^13^^,^^23^. Tratamientos médicos de tipo intravenoso que consideren agentes antirresortivos, quimioterapia o radioterapia, o afectaciones del sistema inmunológico, cardiovascular o trastornos de la coagulación, han sido asociados con un mayor riesgo a complicaciones intra y posoperatorias ^24^. Por lo tanto, existe una relación directamente proporcional entre el estado médico comprometido y pacientes de edades avanzadas, respecto de la contraindicación de este tipo de cirugía. Es decir, a mayor edad y compromiso sistémico, mayor probabilidad de contraindicar el procedimiento quirúrgico y dejar el diente en el lecho óseo, en ausencia de sintomatología y enfermedad. 

La adecuada valoración del terreno biológico y la proximidad que puede existir con estructuras nobles pueden dictaminar la contraindicación de exodoncia de 3Ms, debido a la probabilidad de daño de estructuras adyacentes, como el nervio alveolar inferior ^2^^,^^14^. Las frecuencias de lesiones asociadas a este nervio son bajas; se han reportado en estudios entre un 0,5% y un 5% de las extracciones del 3MI, y se observa un aumento a edades mayores o iguales a 25 años, y con mayor frecuencia en mujeres menores de 30 años ^13^^,^^25^.

Es importante realizar las derivaciones de forma oportuna cuando se requiera, para contribuir a una cobertura adecuada de los sistemas sanitarios. Ante esto, resulta fundamental identificar situaciones clínicas claras de indicaciones o contraindicaciones, siempre bajo el marco de la odontología basada en la evidencia, motivaciones del paciente.

Por último, para futuras investigaciones, sería pertinente contar con estudios de mayor nivel de evidencia incluyendo ensayos clínicos controlados aleatorizados (ECCA) y estudios de cohorte, y realizar un seguimiento de cada caso, según las indicaciones y contraindicaciones de exodoncia de 3Ms. 

## CONCLUSIÓN

Existen situaciones claras para la indicación de exodoncia de 3Ms señaladas en la literatura, las cuales dependen de las características del paciente, la morfología y la posición dentaria. Estas se asocian, principalmente, con cambios patológicos, indicaciones por tratamientos de ortodoncia, cirugía ortognática y tratamientos rehabilitadores.

Por otro lado, la evaluación oportuna de las necesidades y expectativas del paciente, la condición sistémica, el riesgo de morbilidad postquirúrgica, las complicaciones neurosensoriales, el dolor y las implicaciones sociales y financieras, pueden determinar la contraindicación de exodoncia de 3Ms.

Finalmente, en relación con la exodoncia profiláctica, aún existe controversia en la literatura respecto de esta práctica. Sin embargo, se debe considerar en ciertos casos por los beneficios asociados a ella, teniendo en cuenta que no todos los 3Ms asintomáticos y retenidos deben extraerse. Ante esto, el profesional debe ser capaz de evaluar cada caso, anticiparse a los riesgos y valorar los factores que podrían influir en su pronóstico, considerando que esta anticipación también es limitada.

## Fuente de financiamiento:

Autofinanciado
